# Simulated microgravity inhibits C2C12 myogenesis via phospholipase D2-induced Akt/FOXO1 regulation

**DOI:** 10.1038/s41598-019-51410-7

**Published:** 2019-10-17

**Authors:** Mi-Ock Baek, Chi Bum Ahn, Hye-Jeong Cho, Ji-Young Choi, Kuk Hui Son, Mee-Sup Yoon

**Affiliations:** 1 Department of Health Sciences and Technology, GAIHST, Gachon University, Incheon, 21999 Republic of Korea; 2 Lee Gil Ya Cancer and Diabetes Institute, Gachon University, Incheon, 21999 Republic of Korea; 3 Department of Molecular Medicine, School of Medicine, Gachon University, Incheon, 21999 Republic of Korea; 4 Department of Thoracic and Cardiovascular Surgery, Gachon University Gil Medical Center, College of Medicine, Gachon University, Incheon, 21565 Republic of Korea

**Keywords:** TOR signalling, Differentiation, Environmental impact

## Abstract

The skeletal muscle system has evolved to maintain body posture against a constant gravitational load. Mammalian target of rapamycin (mTOR) regulates the mechanically induced increase in the skeletal muscle mass. In the present study, we investigated mTOR pathway in C2C12 myoblasts in a model of mechanical unloading by creating a simulated microgravity (SM) using 3 D clinorotation. SM decreased the phosphorylation of Akt at Ser 473, which was mediated by mTOR complex 2 (mTORC2), in C2C12 myoblasts, leading to a decrease in the cell growth rate. Subsequently, SM inhibited C2C12 myogenesis in an Akt-dependent manner. In addition, SM increased the phospholipase D (PLD) activity by enhancing PLD2 expression, resulting in the dissociation of mSIN1 from the mTORC2, followed by decrease in the phosphorylation of Akt at Ser 473, and FOXO1 at Ser 256 in C2C12 myoblasts. Exposure to SM decreased the autophagic flux of C2C12 myoblasts by regulation of mRNA level of autophagic genes in a PLD2 and FOXO1-dependent manner, subsequently, resulting in a decrease in the C2C12 myogenesis. In conclusion, by analyzing the molecular signature of C2C12 myogenesis using SM, we suggest that the regulatory axis of the PLD2 induced Akt/FOXO1, is critical for C2C12 myogenesis.

## Introduction

Mechanical forces that cause changes in molecular dynamics of cells include stresses and strains on the cell membrane, stretching of the extracellular matrix, or the flow of fluid across the cell surface^1^. The skeletal muscle, being the most mechanosensitive tissue, is highly responsive to mechanical stimuli, including gravitational forces. The skeletal muscle system has evolved to maintain body posture against a constant gravitational load^2^. Both mechanical stimulation and diffusible trophic factors stimulate a pool of myogenic satellite cells which lie beneath the basal lamina and sarcolemma of the muscle, leading to their proliferation, migration to the sites of injury, and fusion with existing muscle fibers^3^. As a result, the biosynthetic capacity is increased during mechanostimulation^3^.

Mechanical unloading such as bed-rest, hind-limb unloading, immobilization, and human spaceflight prevents normal muscle loading, resulting in a decrease in protein synthesis rate and an increase in catabolism causing a significant loss of musculoskeletal mass, size, and strength, and is ultimately followed by muscle atrophy^4^. Exposure of skeletal muscle to microgravity induces alterations in the structural organization of cells, and, as a result, modulates the expression of numerous genes and the activities of numerous enzymes, as well as causing epigenetic modifications^2,5,6^. Hence, deciphering the molecular changes and the process of muscle atrophy that occur following a weightlessness stimulus is required for understanding the mechanism how mechanical unloading elicits muscle atrophy^4^.

Mechanical stimulation has been shown to maintain skeletal muscle mass by regulating protein synthesis^6^. The mammalian target of rapamycin (mTOR), which increases cellular anabolism, especially protein synthesis, and inhibits protein degradation has been known as a critical regulator of protein synthesis in skeletal muscle mass^7^. mTOR forms two biochemically and functionally distinct complexes, mTOR complex 1 (mTORC1) and mTOR complex 2 (mTORC2)^8^. mTORC1 consists of mTOR, the regulatory-associated protein of mTOR (raptor), and G protein β subunit-like (GβL), whereas mTORC2 consists of mTOR, the rapamycin-insensitive component of mTOR (rictor), GβL, mammalian stress-activated protein kinase interacting protein 1 (mSIN1), and Protor 1/2^9^. Both raptor and rictor function as scaffold proteins within mTORC1 and mTORC2, respectively, to facilitate substrate recruitment^10^. Additionally, the activity of mTOR is finely regulated by endogenous inhibitors such as the proline-rich Akt substrate of 40 kDa (PRAS40, which inhibits mTORC1), exchange factor found in platelets, leukemic, and neuronal tissues (XPLN, which inhibits mTORC2), and DEP domain containing mTOR interacting protein (DEPTOR, which inhibits mTORC1 and mTORC2), suggesting that the composition of the mTOR complex is important for maintaining mTOR activity^10^. mTORC1 is activated by growth factors, cellular energy levels, oxygen status, and amino acids, and leads to the stimulation of protein synthesis through the phosphorylation of two key effectors, ribosomal protein S6 kinase 1 (p70S6 kinase 1;S6K1) and eIF4E binding protein1 (4EBP1)^8^. On the contrary, mTORC2 functions as an effector of insulin/phosphoinositide 3-kinase (PI3K) signaling by regulating protein kinase B (PKB, also known as Akt), glycogen synthase kinase (GSK), and protein kinase C (PKC). Among the mTORC2 components, mSIN1 is critical for modulating mTORC2 activity through auto-inhibition of the PH domain, which is relieved by phosphatidylinositol (3,4,5)-triphosphate (PIP3) and phosphorylation by Akt, resulting in an enhancement of mTORC2 kinase activity^11^.

Mechanical stimuli have been shown to increase phosphatidic acid (PA) levels, which subsequently activate mTOR and, in turn, promote protein synthesis in skeletal muscle^12^. mTOR is known to be an essential regulator of mechanical-induced cellular responses, especially an increase in protein synthesis rate and muscle mass; however, the molecular mechanisms through which mTOR is activated by mechanical stimulation have not been well defined. Although simulated microgravity (SM) is a model system for the effect of mechanical unloading on biological system and mTOR is a critical regulator of myogenesis, fusion, and muscle regeneration, the role of mTOR under SM conditions is not fully understood.

In the present study, we have achieved SM using 3D clinorotation. We demonstrated that the exposure of C2C12 myoblasts to SM decreased Akt phosphorylation and triggered a defect in cell growth, subsequently resulting in poor myogenic differentiation in an Akt-dependent manner. Our results strongly suggested that there is an increase in PLD2 expression following exposure to SM conditions that resulted in the dissociation of mSIN1 from the mTORC2 complex, followed by decreased Akt phosphorylation and increased dephosphorylation of FOXO1. Subsequently, the expression of autophagic genes was increased, whereas autophagic flux was blocked. Taken together, we have reported for the first time that the molecular signature of PLD2/Akt/FOXO1/autophagic flux under SM conditions is critical for early phase of myogenesis in C2C12 cells.

## Results

### SM inhibits Akt phosphorylation at Ser473 in C2C12 myoblasts

**Figure 1:**
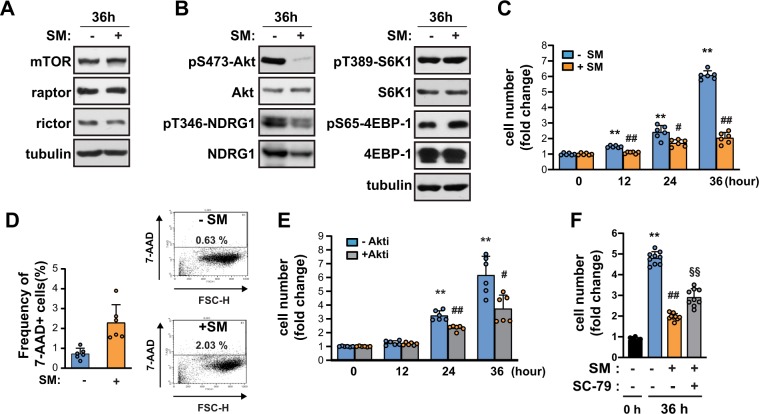
SM inhibits the growth of C2C12 myoblast by blocking pS473-Akt. (**A,B**) C2C12 myoblasts were incubated either under terrestrial gravity (1 *g*) or under SM for 36 h, lysed and subjected to western blotting. (**C**) The cells were treated as in (**A**) for the indicated time and tryphan blue stained cells were counted using a cell counter (n = 6). (**D**) The cells were treated as in (**A**), stained with 7-AAD, and analyzed by flow cytometry (n = 6). (**E**) The cells were incubated with or without 1 μM Akti for indicated time and counted (n = 6). (**F**) The cells were incubated under SM in either presence or absence of 10 μM SC79 for 36 h and counted (n = 9). Abbreviations: simulated microgravity (SM); 7-aminoactinomycin D (7-AAD); Akt inhibitor (Akti). All blots shown are representative of 3 independent experiments. Data are expressed as mean ± SD, with Mann-Whitney U test performed as indicated. **P < 0.01, *P < 0.05 versus control at 0 h; ^##^P < 0.01, ^##^P < 0.01 versus control at each indicated time; ^§§^P < 0.01 versus SM-exposed cell for 36 h.

To understand how a reduced gravitational force induces muscle wasting, we first examined whether microgravity affects mTOR signaling in myoblasts using a clinostat which is widely used to generate SM. C2C12 myoblasts were grown to 80–90% confluence, and then placed in either a stationary control (1 *g*) or in the dynamic reactor to simulate 0 *g* by rotating in both the vertical and horizontal planes. The expression levels of mTOR, raptor, or rictor in C2C12 cells were not changed under SM condition (Fig. 1A). Next, to further investigate any potential changes in mTOR kinase activity, we examined the phosphorylation of several downstream targets of mTOR after the C2C12 myoblasts were exposed to SM for 36 h. After the exposure to SM, mTORC2-mediated Akt phosphorylation at Ser 473 (pS473-Akt) and NDRG1 phosphorylation at Thr346 were decreased (Fig. 1B). On the other hand, the mTORC1-mediated phosphorylation of S6K1 and 4EBP1 remained unchanged (Fig. 1B). To address the functional relevance of the decrease of pS473-Akt levels on the growth of myoblasts, the number of C2C12 cells was measured at 12, 24, and 36 h of either SM or terrestrial gravity conditions. Myoblasts clearly grew more slowly under SM conditions (a 3.5-fold reduction was seen at 36 h) (Fig. 1C). The percentage of dead cells was comparable in both SM and terrestrial gravity conditions, as shown by the frequencies of 7-aminoactinomycin (7-AAD) + cells (Fig. 1D), which suggests no difference in viability between both conditions. Treatment of the myoblasts with Akti, an Akt kinase inhibitor, decreased the cell growth rate significantly (Fig. 1E), which mimicked the growth retardation of cells under SM conditions. In addition, treatment of the cells exposed to SM with SC79, an Akt activator, restored the growth rate of C2C12 myoblasts (Fig. 1F). These results suggested that Akt inhibition causes the growth inhibition in C2C12 myoblasts under SM condition.

### Pretreatment with SM inhibits myogenesis in C2C12 cells in an Akt-dependent manner

**Figure 2:**
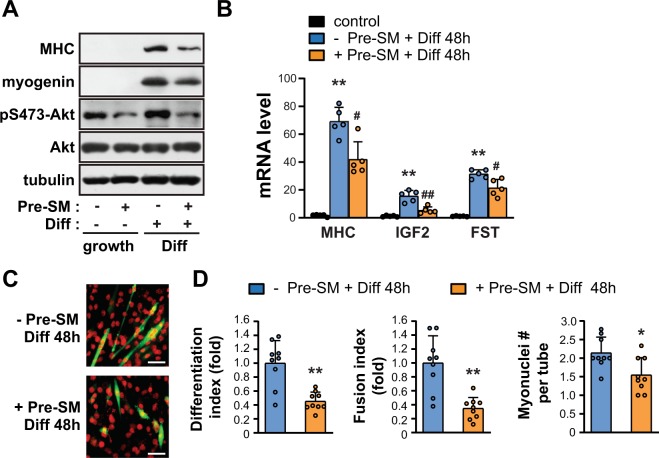
Pretreatment with SM inhibits myogenesis. C2C12 myoblasts were incubated either under 1 *g* or under SM for 24 h, after which they were induced to differentiate under 1 *g* for 48 h. (**A**) Cells were lysed and subjected to western blotting. (**B**) Quantitative RT-PCR was performed to analyze the relative levels of MHC, IGF2, and FST. Mouse GAPDH was used to normalize gene expression (n = 5). **P < 0.01 versus undifferentiated control; ^##^P < 0.01, ^#^*P* < 0.05 versus differentiated control without pre-SM. (**C**) The cells were stained for MHC (green) and DAPI (red). scale bar = 50 μm. (**D**) The differentiation index (differentiated nuclei/total nuclei), fusion index (fused nuclei/total nuclei), and average myotube size (number of nuclei/myotube) were quantified (n = 9). **P < 0.01, *P < 0.05 versus differentiated control without pre-SM. Abbreviations: simulated microgravity (SM); differentiation (Diff); myosin heavy chain (MHC); insulin-like growth factor 2 (IGF2); follistatin (FST); simulated microgravity for 24 h before the induction of differentiation (Pre-SM). Data are expressed as mean ± SD, with Mann-Whitney U test performed as indicated.

**Figure 3:**
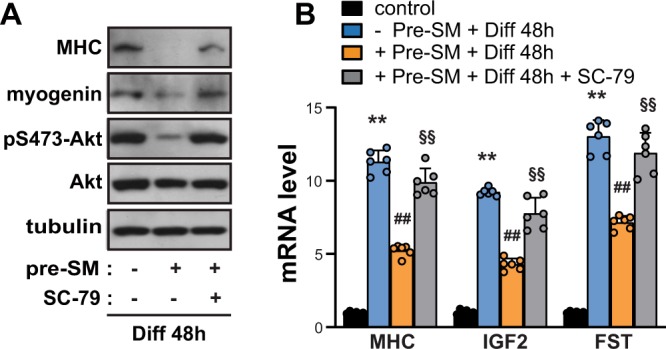
SC79 rescued myoblast from SM-induced inhibition of C2C12 myogenesis. C2C12 myoblasts were incubated under SM in either the presence or absence of 10 μM SC79 for 24 h, and then were induced to differentiate under 1 *g* for 48 h. (**A**) Cells were lysed and subjected to western blotting. (**B**) Quantitative RT-PCR was performed to analyze the relative levels of MHC, IGF2, and FST. Mouse GAPDH was used to normalize gene expression (n = 6). **P < 0.01 versus undifferentiated control; ^##^P < 0.01 versus differentiated control without pre-SM; ^§§^P < 0.01 versus differentiated control with pre-SM. Abbreviations: simulated microgravity (SM); differentiation (Diff); myosin heavy chain (MHC); insulin-like growth factor 2 (IGF2); follistatin (FST); simulated microgravity for 24 h before the induction of differentiation (Pre-SM). Data are expressed as mean ± SD, with Mann-Whitney U test performed as indicated.

During myogenesis, myoblasts must exit the cell cycle and subsequently undergo an ordered set of myogenic events, such as migration, cell-to-cell adhesion, and myotube fusion^13^. It was of interest as to how myogenic differentiation was affected by SM on C2C12 myoblasts. To test this, C2C12 myoblasts were grown under SM conditions for 24 h, and then serum was withdrawn for 48 h to induce differentiation. As a result, C2C12 myoblasts differentiated following serum withdrawal, as indicated by the expression of the early myogenic marker myogenin and the late myogenic marker myosin heavy chain (MHC) over the course of 2 days (Fig. 2A). When the level of pS473-Akt was decreased in SM-pretreated myoblasts and myocytes (Fig. 2A), myogenic differentiation of SM-pretreated cells was significantly suppressed, as shown by the reduced myogenin and MHC levels (Fig. 2A). Consistent with this, the mRNA levels of MHC, and myogenic markers such as insulin-like growth factor 2 (IGF2) and follistatin (FST) were reduced (Fig. 2B). Both the formation and the fusion of myotubes were suppressed in the pre-SM exposed cells, as evidenced by quantification of the differentiation index, the fusion index, and average myotube size (marked by myonuclei number per myotube) (Fig. 2C,D). In addition, treatment of C2C12 myoblasts under SM condition with SC79, an Akt activator, rescued SM-induced inhibitory effects on C2C12 differentiation, as proved by restoration of protein level of MHC and myogenin (Fig. 3A) and mRNA level of MHC, IGF2, and FST (Fig. 3B). These results suggested that the Akt inactivation that occurs following pretreatment with SM leads to a defect in myogenic differentiation.

### SM suppresses C2C12 myogenic differentiation at an early phase

**Figure 4:**
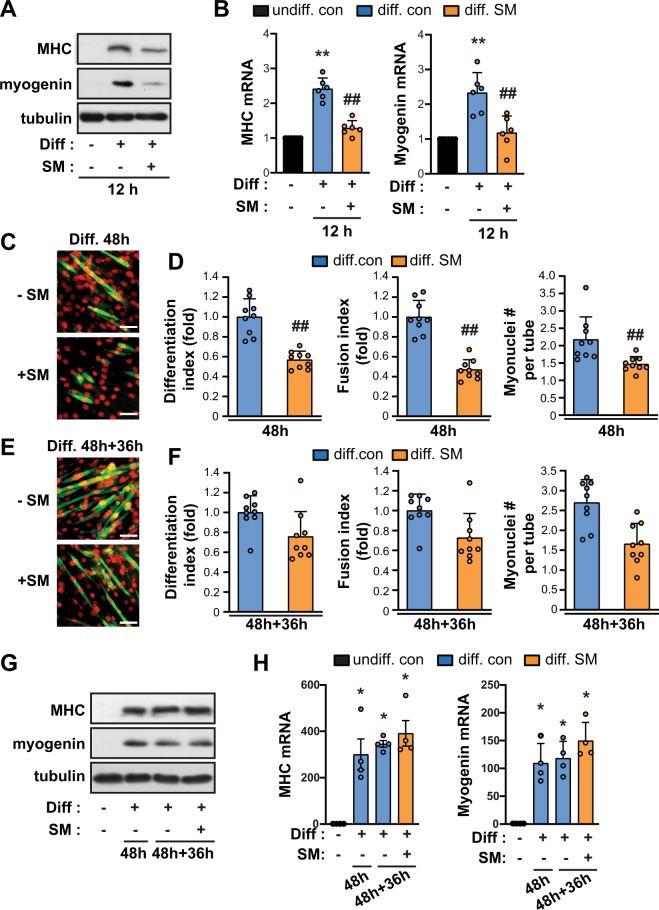
SM suppresses C2C12 myogenic differentiation at an early phase of myogenesis. (**A,B**) C2C12 myoblasts were induced to differentiate either under 1 *g* or SM for 12 h. Cells were lysed and subjected to western blotting (**A**) or a quantitative RT-PCR analysis of MHC and myogenin expression (n = 6) (**B**). **P < 0.01 versus undiff. con; ^##^*P* < 0.01 versus diff. con. (**C**,**D**) The cells were differentiated either under 1 *g*, or under SM for 48 h. (**C**) The cells were stained for MHC (green) and DAPI (red). scale bar = 50 μm (**D**) The stained images were quantified as described in Fig. 2C (n = 9). ^##^*P* < 0.01 versus diff.con. (**E**–**H**) C2C12 myoblasts were differentiated for 48 h and shifted either to 1 *g* or SM for 36 h. (**E**) The cells were stained for MHC (green) and DAPI (red). scale bar = 50 μm. (**F**) The quantification of images was determined as described in Fig. 2C (n = 9). (**G**,**H**) Cells were lysed and subjected to western blotting (**G**) or a quantitative RT-PCR analysis of MHC and myogenin expression (n = 4) (**H**). *P < 0.05 versus undiff. con. Mouse GAPDH was used to normalize gene expression. Abbreviations: simulated microgravity (SM); differentiation (Diff); undifferentiated control (undiff. con); differentiated control (diff. con); differentiated under SM condition (diff.SM). Data are expressed as mean ± SD, with Mann-Whitney U test performed as indicated.

To further address the effect of SM on the initiation of myogenic differentiation, we induced myogenic differentiation by serum withdrawal, with or without exposure to the SM condition. The levels of myogenin mRNA and protein were completely suppressed under SM conditions, accompanied by dampening of MHC expression levels (Fig. 4A,B), resulting in a quantifiable reduction in myotube formation (Fig. 4C) as quantified by the differentiation index, the fusion index, and myotube size (Fig. 4D). When C2C12 myoblasts were differentiated for 2 days under 1 *g* and then shifted to SM conditions for 36 h, the myotubes that formed were smaller than those in the differentiation control, but the changes in the differentiation index or the fusion indices were not statistically significant (Fig. 4E,F). Neither the mRNA nor the protein levels of MHC and myogenin were changed significantly (Fig. 4G,H), suggesting that exposure to SM during the late phase of differentiation does not significantly affect either myotube fusion or differentiation. These results suggest that SM blocks myogenic differentiation at both the proliferative phase, and in particular at the early phase of differentiation.

### SM increases PLD2-induced PA, leading to dissociation of mSIN1 from mTORC2 and a reduction of pS473-Akt in C2C12 myoblasts

**Figure 5:**
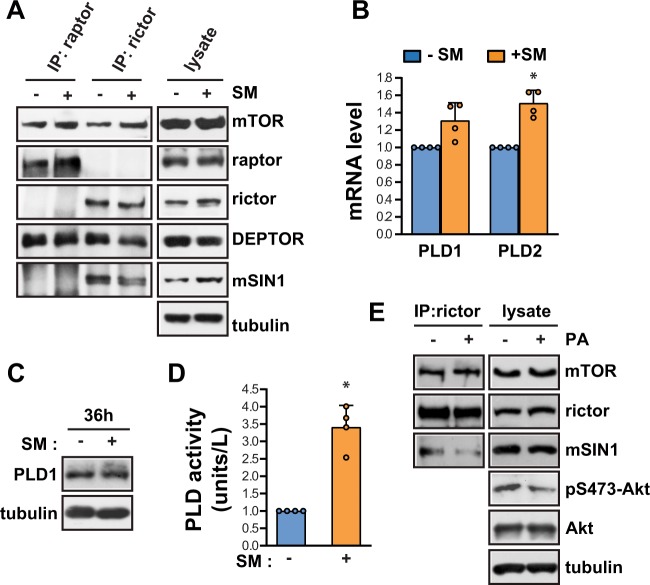
SM decreases mSIN1 levels in mTORC2 by PLD2-induced PA production in C2C12 myoblasts. (**A**) C2C12 myoblasts were incubated either under terrestrial gravity (1 *g*) or under SM for 36 h, lysed, and subjected to immunoprecipitation using antibodies against either raptor or rictor, and analyzed by western blotting. (**B**) Cells were treated as in (**A**), lysed, and subjected to quantitative RT-PCR for PLD1 and PLD2 (n = 4). (**C**) Cells were treated as in (**A**), lysed, and analyzed by western blotting. (**D**) PLD assay was performed using a PLD assay kit (n = 4). (**E**) Cells were treated with or without 300 μM C8-PA for 30 min, lysed, then subjected to immunoprecipitation with antibody against rictor, and analyzed by western blotting. Abbreviations: simulated microgravity (SM); immunoprecipitate (IP); Phosphatidic acid (PA). All data are blots representative of 3 to 5 independent experiments. Data are expressed as mean ± SD, with Mann-Whitney U test performed as indicated. *P < 0.05; versus control under 1 *g*.

Since the SM-induced defect in Akt phosphorylation results in decreased C2C12 myogenic differentiation, we set out to investigate the mechanism by which SM decreases pS473-Akt levels. First, we set out to examine whether SM affects the composition of the mTORC2 complex, which is critical for mTORC2 kinase activity. We isolated mTORC2 using an antibody against rictor and analyzed the components of mTORC2 by western blot analysis. Notably, treatment with SM for 36 h decreased the level of mSIN1 in the mTORC2 complex, without any changes in the levels of rictor or mTOR (Fig. 5A). In contrast to these changes in mTORC2, the levels of raptor, mTOR, and DEPTOR remained unchanged in mTORC1 by immunoprecipitation using antibody against raptor (Fig. 5A), consistent with the lack of change in mTORC1 activity demonstrated by the normal phosphorylation of mTORC1 targets, such as S6K1 and 4EBP1 (Fig. 1B). Phosphatidic acids (PAs) are known to regulate both the stability and activity of mTOR complexes and bind to the FKBP12-rapamycin binding (FRB) domain of mTOR to activate mTORC1 kinase activity by displacing DEPTOR, an endogenous mTOR inhibitor^14,15^. Previously, phospholipase D (PLD) 1, an isoform of PLD which produces PA, has been reported to be a positive regulator of myogenesis in C2C12 cells^16^ as well as muscle regeneration^17^. We wondered whether SM affected PLD activity, and whether this had any effect on mTOR complex assembly. Under SM conditions, the mRNA (Fig. 5B) and protein levels (Fig. 5C) of PLD1 remained unchanged, however, the levels of PLD2 mRNA expression increased significantly (Fig. 5B). PLD2 protein levels could not be detected directly because of the lack of a good commercially available anti-PLD2 antibody^18^. Consistent with this, PLD activity was enhanced up to 3.5-fold compared to cells exposed to terrestrial gravity (Fig. 5D). Moreover, PA treatment significantly decreased mSIN1 levels in mTORC2, as well as pS473-Akt levels in C2C12 myoblasts (Fig. 5E), suggesting that the PLD2-mediated production of PA suppresses pS473-Akt levels by reducing the mSIN1 levels, and thereby the kinase activity, of mTORC2.

### SM regulated autophagic flux in an PLD2 and FOXO1-dependent manner

**Figure 6:**
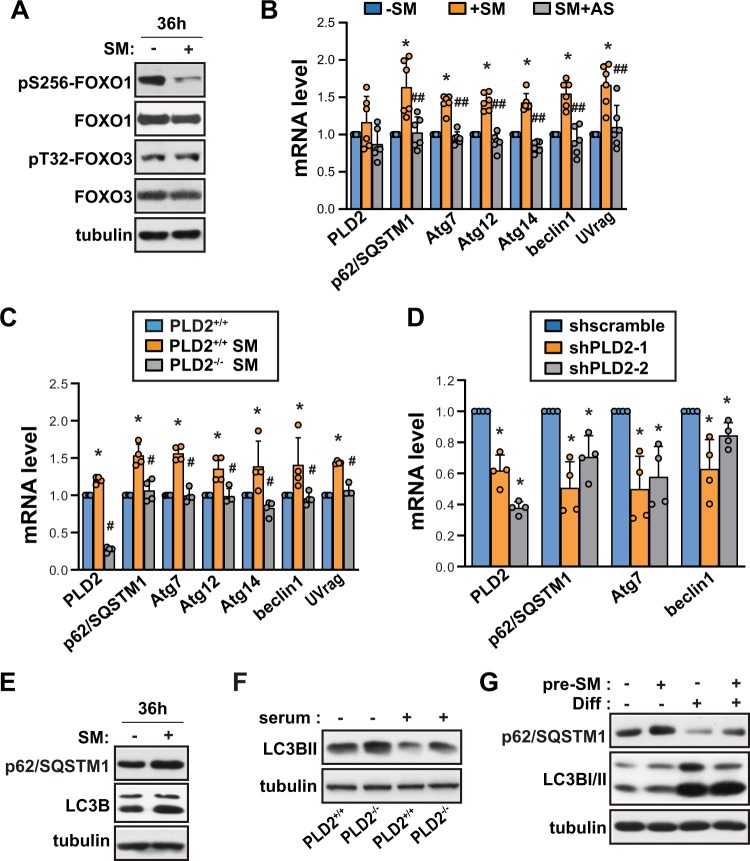
SM regulated autophagic flux in an PLD2 and FOXO1-dependent manner. (**A**) C2C12 myoblasts were incubated either under terrestrial gravity (1 *g*) or under SM for 36 h, lysed, and analyzed by western blotting. (B) Cells were treated as (**A**), with or without 100 nM AS1842856, lysed and subjected to quantitative RT-PCR (n = 6). (**C**) Either PLD2^+/+^ MEFs or PLD2^−/−^ MEFs were incubated either under terrestrial gravity (1 *g*) or under SM for 36 h, lysed, and analyzed by quantitative RT-PCR (n = 4). (**D**) The cells were transduced with lentiviruses expressing shRNAs against PLD2 (shPLD2-1, shPLD2-2) or a scrambled sequence as control, selected with puromycin for 3 days, lysed, and analyzed by quantitative RT-PCR (n = 4). (**E**) Cells were treated as (**A**), lysed, and analyzed by western blotting. (**F**) Either PLD2^+/+^ MEFs or PLD2^−/−^ MEFs were incubated with or without serum for 18 h, lysed, and subjected to western blotting. (**G**) C2C12 myoblasts were incubated either under 1 *g* or under SM for 24 h, after which they were induced to differentiate under 1 *g* for 48 h. The cells were lysed and subjected to western blotting. Abbreviations: simulated microgravity (SM); simulated microgravity in the presence of 100 nM AS1842856 (SM + AS); differentiation (Diff); simulated microgravity for 24 h before the induction of differentiation (pre-SM). All are blots representative of 3 to 5 independent experiments. Data are expressed as mean ± SD, with Mann-Whitney U test performed as indicated. **P < 0.01, *P < 0.05 versus 1 *g* control; ^##^*P* < 0.01, ^#^*P* < 0.05 versus simulated microgravity.

Forkhead box O (FOXO) factors are phosphorylated by Akt, which promotes the translocation of FOXO factors from the nucleus to the cytosol and subsequently reduces FOXO transcriptional activity^19^. In contrast, dephosphorylated FOXO has enhanced FOXO transcriptional activity and results in decreased myocyte size through the induction of atrophy- and autophagy-related genes^20,21^. FOXO1 phosphorylation at Ser 256, an Akt target site, was found to be decreased following exposure of cells to SM conditions for 36 h (Fig. 6A), consistent with the decrease in pS473-Akt levels (Fig. 1B). On the contrary, FOXO3 phosphorylation at Thr 32 remained unchanged (Fig. 6A), suggesting that FOXO1, rather than FOXO3, is mainly involved in SM-induced signaling in C2C12 myoblasts. FOXO1 activation has been shown to be associated with an induction of autophagy-related gene expression in cardiomyocytes^22^. As shown in Fig. 6B, autophagy-related genes which are involved in autophagosome formation (beclin1, UVrag, p62/SQSTM1, Atg7, Atg14) and autophagosome maturation (Atg12), were increased following exposure to SM conditions. Treatment with AS1842856, a FOXO1-specific inhibitor, significantly suppressed the mRNA expression of autophagy-related genes under SM conditions (Fig. 6B). In addition, to test the effect of PLD2 depletion on mRNA expression levels of autophagy-related genes, we used mouse embryonic fibroblasts (MEFs) from previously reported PLD2^−/−^ mice^23^, and depleted PLD2 in C2C12 myoblasts using two different shRNAs. mRNA expression levels of autophagy-related genes remained unchanged under SM conditions in PLD2-depleted MEF (Fig. 6C), suggesting that autophagy-related genes were expressed in a PLD2-dependent manner in response to SM. Consistent with this, depletion of PLD2 using two different shRNAs in undifferentiated C2C12 myoblasts reduced the expression of autophagy-related genes (Fig. 6D). However, despite there being an increase in autophagic genes, autophagic flux was blocked in SM-exposed C2C12 myoblasts, as evidenced by the increased LC3BII/LC3BI ratio and the accumulation of p62/SQSTM1 (Fig. 6E). Autophagic flux was increased in PLD2−/− MEFs in either the presence or absence of serum, as evidenced by the increase in LC3BII level (Fig. 6F). Next, to examine whether the suppression of autophagic flux under SM conditions regulates myogenesis, we analyzed autophagic flux in C2C12 cells after the induction of myogenesis with or without SM-pretreatment. Autophagic flux was found to be increased in differentiated myocytes (Fig. 6G), as has previously been reported^24^. Pretreatment with SM suppressed autophagic flux as shown by increase in p62/SQSTM1 and decrease in LC3BII (Fig. 6G), which was accompanied by a defect in differentiation as indicated by the reduced expression of MHC and myogenin, and by the differentiation index (Fig. 2A–D). Taken together, these results suggest that SM suppresses autophagic flux and myogenic differentiation in a PLD2 and FOXO1-dependent manner.

## Discussion

**Figure 7:**
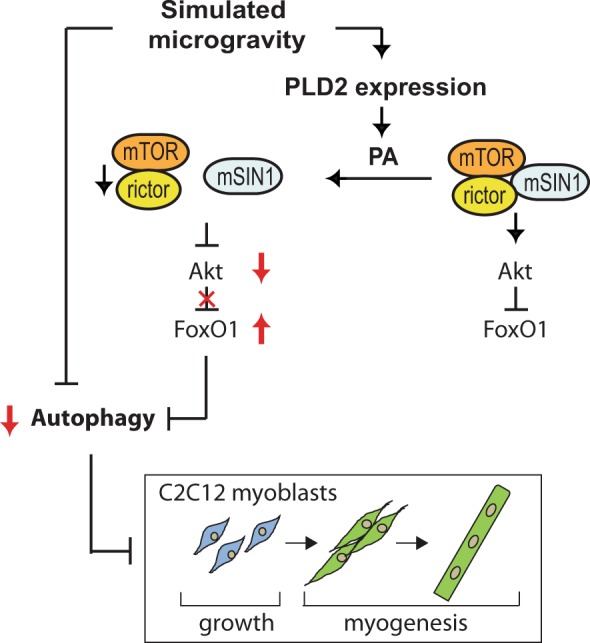
A proposed model of regulation of the PLD2/Akt/FOXO1 axis in C2C12 myoblasts under simulated microgravity (SM). Treatment with SM in C2C12 myoblasts augments PLD2 expression/PA level, leading to the dissociation of mSIN1 from the mTORC2 signaling complex. Subsequently, Akt phosphorylation is reduced, followed by FOXO1 activation as well as a reduction in autophagic flux through an increase in the expression of autophagy-related genes. These cellular events dampen the growth of C2C12 myoblasts, as well as the differentiation of C2C12 myocytes.

Mechanical signaling plays a crucial role in determining the skeletal muscle mass^7^. In the present study, we report that mechanical stimulation is critical for C2C12 myogenesis in a model of mechanical unloading using SM induced by a clinostat. The constant rotation of a sample induces the time average for the *g* vector to be close to 0 *g*^25^, which is able to provide a condition representative of mechanical unloading. We provide the first evidence of a cellular response in C2C12 myogenesis under SM conditions that leads to a reduction in Akt phosphorylation and, subsequently, FOXO1 activation, as well as a reduction in autophagic flux through an increase in the expression of autophagy-related genes (Fig. 7). We also proved that the mechanism of Akt inactivation occurs through the PLD2-mediated production of PA which induces the dissociation of mSIN1 from the mTORC2 signaling complex (Fig. 7).

The present study identifies a distinct role for mTORC2, separate from mTORC1, in mechanical unloading. We firstly discovered that the decreased levels of mSIN1 in mTORC2 led to a significant decrease in the levels of pS473-Akt, an mTORC2 kinase activity biomarker, under SM conditions. A decrease in Akt phosphorylation has been shown to play a role in the mechanical adaptation of the cytoskeleton in mesenchymal stem cells to regulate mechanical lineage decisions^26^. Notably, PA induced the dissociation of mSIN1 which was critical in regulating mTORC2 activity under SM conditions. Glycerol-3-phosphate acyltransferase-1 (GPAT1)-produced PA has also been shown to inhibit mTORC2 activity, not by displacing mSIN1 but by preferentially disrupting the binding between mTOR and rictor^27^. PLD2 is primarily localized to the plasma membrane, unlike PLD1 which is present at the lysosome, Golgi-complex, and the endosome^28^. Both PLD1 and PLD2 hydrolyze a structurally similar substrate pool and produce identical PA species enriched in mono- and di-unsaturated fatty acids^29^. Hence, we could speculate that PLD2 forms a PA pool near the cell plasma membrane, which is separated from the PLD1-produced PA pools in the lysosome and the endosome. Ebner *et al*. showed that the activity and localization of mTORC2 via Sin1 at the plasma membrane is both PI3K- and growth factor-independent, suggesting the existence of spatially separated mTORC2 populations, and the importance of mTORC2 localization to the plasma membrane for Akt activation^30^. In the present study, the increase of PLD activity under SM conditions was attributed to changes in PLD2 expression due to the absence of significant changes in PLD1 expression. Hence, we propose that PLD2 forms a spatially separate PA pool at the plasma membrane that regulates mTORC2 activity through mSIN1.

Previous reports have shown that the FOXO protein family members FOXO1 and FOXO3 promote autophagy. In muscle, activated FOXO3 has been shown to induce the expression of numerous autophagy-related genes, which are also expressed in atrophying muscle^21^. However, in the present study, FOXO1 phosphorylation was suppressed under SM conditions without any change in FOXO3 phosphorylation (Fig. 6A). FOXO1 has been shown to induce autophagy-related genes transcriptionally^31^. Surprisingly, the increase in autophagy-related genes induced by FOXO1 worsened the defect in autophagic degradation in C2C12 cells under SM conditions. Cytoplasmic FOXO1 dissociates from sirtuin-2 (SIRT2), a deacetylase, is then acetylated, and binds to Atg7 to promote autophagy and apoptosis under conditions of serum starvation or oxidative stress^32^. Hence, we speculate that the majority of the dephosphorylated FOXO1 relocalizes to the nucleus under SM conditions, resulting in a depletion of the pool of cytoplasmic FOXO1 and a blockage of FOXO1-Atg7 mediated autophagy, which then leads to a defect in autophagic flux. Whether SM changes the distribution of FOXO1 and subsequent inhibition of autophagic flux warrants further investigation.

In this study, we showed that autophagic flux was impaired in C2C12 myoblasts under SM conditions, followed by a defect in both myogenic differentiation and myogenic fusion. The inducible activation of a FOXO1 active mutant in C2C12 myoblasts has been reported to impede myogenic differentiation at an early stage and simultaneously lead to the proteasome-dependent degradation of mTOR and raptor^33^. However, in the present study, the expression of both mTOR and raptor remained unchanged (Fig. 1A), which led us to speculate that FOXO1 under SM conditions plays a critical role in the regulation of autophagy instead of protein degradation. Autophagy is an early event in myogenic differentiation, which is crucial for the initiation of myogenic differentiation, cargo trafficking, and the lysosomal fusion step^24^. In addition, depletion of autophagy augmented muscle loss during denervation and fasting, implying the importance of autophagic flux in maintenance of the muscle mass and myofiber intergrity^34^. Notably, myotube formation was delayed in SM-pretreated myoblasts, suggesting that blocking autophagic flux in myoblasts inhibits myogenic differentiation under SM conditions. Since p53-dependent induction of autophagy and the remodeling of the mitochondrial network through mitophagy is essential for myogenic differentiation^24,35^, further examination of the involvement of either p53 or mitophagy is needed to further dissect autophagy during C2C12 myogenesis.

The skeletal muscle inactivity for long periods of time or mechanical unloading of bed rest, hindlimb unloading, immobilization, and spaceflight has been used to investigate loss of musculoskeletal mass, size, and strength^4^. The characteristics of the skeletal muscles under microgravity have been shown to be similar to them with low load on terrestrial gravity. Exposure to microgravity in space declines in skeletal muscle size, volume, central surface area (CSA), strength significantly, even long-term exposure to microgravity exacerbates the changes in skeletal muscles^36,37^. Further short-exposure to microgravity induces the shift of soleus muscle toward a faster glycolytic phenotype^38^, whereas it has no effects on fiber type composition in fast-twitch muscles^39,40^. The decrease in protein synthesis of muscles, which Akt-mTOR pathway plays a role in^41^, has been recognized as the major cause of disuse muscle atrophy. In addition, reduction of mTOR activity inhibit regrowth of muscle during the recovery from hindlimb immobilization without affecting protein degradation^42^. However, mechanical unloading reduces the phosphorylation of Akt and glycogen synthase kinase-3β (GSK-3β) in association with impaired ribosome biogenesis in an mTORC1 independent manner^43^. In the current study, we also observed the decline in phosphorylation of Akt, but not in mTORC1, under SM condition, subsequent dampening of C2C12 myogenic differentiation, implying the critical role of Akt in muscle differentiation under SM condition. Since GSK-3β regulates glycogen metabolism and protein turnover of skeletal muscles as well as mitochondrial biogenesis during myogenesis^44^, further investigation is required to test the role of GSK-3β in C2C12 myogenesis under SM condition.

However, in spite of the similarity between spaceflight and the other mechanical unloading methods on effects on muscles, the transcriptome profiles subjected to microgravity reported to show minor similarities with a publicly available data set of hindlimb suspension which is a way to induce disuse muscle atrophy^45^. It suggested that loss of muscle mass and strength by exposure to microgravity is unique when compared to the loss of muscle mass and strength by hindlimb suspension^45^. Thus, the present study to investigate the regulatory mechanism of C2C12 myogenesis under SM condition is worth in light of mimicking the changes in the skeletal muscles in space, distinct from ones of other atrophic conditions.

## Methods

### Antibodies and other reagents

Antibodies were obtained from the following sources: anti-raptor, -rictor, and -mSIN1 antibodies were from Bethyl Laboratories (Montgomery, TX, USA); MF20 anti-sarcomeric MHC and F5D anti-myogenin antibodies were from the Developmental Studies Hybridoma Bank (developed under the auspices of the National Institute of Child Health and Human Development, National Institutes of Health, and maintained by the Department of Biological Sciences, University of Iowa, Iowa City, IA, USA); anti-DEPTOR were from Novus (Littleton, Colorado, USA). All other primary antibodies were from Cell Signaling Technology (Danvers, MA, USA). All secondary antibodies were from Jackson ImmunoResearch Laboratories Inc. (West Grove, PA, USA). 1,2-dioctanoyl-sn-glycero-3–PA was obtained from Avanti Polar Lipids (Alabaster, AL, USA). Akti1/2 were from Calbiochem-Merck (Darmstadt, Germany). Mouse PLD2 shRNA were previously described^16^. All other reagents were from Sigma-Aldrich (St. Louis, MO, USA).

### Cell culture

C2C12 myoblasts were maintained in Dulbecco’s modified Eagle’s medium (DMEM) containing 4.5 g/L glucose with 10% fetal bovine serum (FBS) at 37 °C in an atmosphere of 5% CO_2_. Cells were plated 18 h before being placed under microgravity conditions in a polystyrene (PS)/polyolefin based cell culture dish plate (SPLPermea^™^, SPL Life Sciences Co., Gyeonggi-do, Korea). To induce differentiation, C2C12 cells were plated on tissue culture plates coated with 0.2% gelatin and grown to 100% confluence before the media was changed to differentiation medium (DMEM containing 2% horse serum). Cultures were replenished with fresh medium daily for 3 days. To test the effect of SM on the initiation of differentiation, cells were induced to differentiate under SM conditions, and then harvested at the indicated times after the induction of differentiation. To test the effect of SM on the fully differentiated cells, cells were differentiated for 3 days, and then incubated under SM conditions for 18 h. PLD2^+/+^ and PLD^−/−^ MEFs were prepared as described previously^23^. All experimental protocols for animals, maintenance and care, were conducted according to Gachon University Animal Care guidelines. All animal procedures were approved by the Center of Animal Care and Use, Lee Gil Ya Cancer and Diabetes Institute, Gachon University and the Institutional Animal Care and Use Committee (IACUC).

### SM: the clinostat system

A clinostat system (3D clinostat, Shamhantech Inc., Bucheon, Korea), known as an effective system for simulating microgravity on the ground, was used in this study. This system is composed of two rotational axes and the rotation speed of each axis was fixed to 5 rpm (0.523 rad/s). These axes controlled each motors and drivers set (AiS-42MA, Autonics, Busan, Korea) (Suppl. Fig. S1A). The centrifugal accelerations of the system calculated by Eq. (1) were 0.0047 G (edge) to 0 G (center)^46,47^.

1 $${a}_{c}=r{\omega }^{2}=\frac{r{\omega }^{2}}{9.8}G$$

$$({F}_{c}=m{a}_{c}=mr{\omega }^{2},\,1G=9.8(m/{s}^{2})$$

where a_c_ is the centripetal acceleration generated by rotating system (m/s^2^), ω is the angular velocity(rad/s), r is the distance of the sample to the center of rotation (m), G is acceleration of gravity, F_c_ is the experienced centripetal force (N) and m is mass (kg). C2C12 cells were plated in a PS/polyolefin based cell culture dish (SPLPermea^™^, SPL Life Sciences Co.) (Suppl. Fig. S1B). The cell culture dish is round (44.88 × 12.65 mm, diameter × height), and its top is porous to air. After the cells were attached to the cell culture dish, the dish was filled with culture medium (maximum volume; 10 ml). To avoid air bubbles in the culture dish, the top cover of culture dish was closed in the flask which contained cell media when culture media was filled in the culture dish. The absence of air bubbles after sealing the culture dishes was confirmed every time. The plate was completely sealed during the rotation on the clinostat. The dish was fixed carefully to the rotating panel of the clinostat system, which was then placed in an incubator at 37 °C with a 5% CO_2_ atmosphere (Suppl. Fig. S1C). The clinostat was continuously rotated at 5 rpm for 36 h. The control cells (normal gravity) were plated on the same type of dish (SPLPermea^™^, SPL Life Sciences Co.) and incubated in the same incubator as the cells exposed to the SM, but did not undergo clinorotation (Suppl. Fig. S1C).

### Cell proliferation and viability

Cell counting was performed using a cell counter (LUNA-II™ Automated Cell Counter, Gyenggi-do, Korea) and the number of trypan blue (WEL GENE, Gyeongsangbuk-do, Korea)-stained cells was used to assess cell growth rate, according to the dye exclusion method^48^. All counts were performed in duplicate with 6 independent samples after 12, 24, and 36 h of growth. For flow cytometry analysis (BD FACS Calibur, BD Science, San Jose, CA, USA), cells were collected at a concentration of 1.5 × 10^5^ cells/tube, and incubated with 7-AAD for 5 min on ice in the dark.

### Cell lysis, immunoprecipitation, and western blot analysis

Cells and tissues were lysed in ice-cold lysis buffer (Cell Signaling Technology, Danvers, MA, USA) containing a protease inhibitor cocktail (Sigma-Aldrich, St. Louis, MO, USA). The supernatant was collected after microcentrifugation at 13,000 × *g* for 10 min, and then either used for immunoprecipitation or boiled in sodium dodecyl sulfate (SDS) sample buffer for 3 min. Immunoprecipitation was performed with anti-raptor or anti-rictor antibodies, followed by incubation with protein G agarose for 1 h at 4 °C. For immunoprecipitation of raptor, lysis buffer containing 40 mM 4-(2-hydroxyethyl)-1-piperazineethanesulfonic acid (pH 7.4), 120 mM NaCl, 10 mM pyrophosphate, 50 mM NaF, 10 mM β-glycerophosphate, 2 mM EDTA, 1 × Sigma protease inhibitor cocktail, and 0.3% 3-[(3-cholamidopropyl) dimethylammonio]-1-propanesulfonate was used. The beads were washed three times with lysis buffer and then boiled in SDS sample buffer for 3 min. Proteins were separated by electrophoresis on SDS-polyacrylamide gels, and then transferred onto polyvinylidene fluoride membranes (Millipore, Billerica, MA, USA). Antibody incubations were performed using the manufacturer’s recommended conditions. Horseradish peroxidase-conjugated secondary antibodies were detected with Immobilon Western Chemiluminescent HRP Substrate (Millipore, Billerica, MA, USA).

### RNA isolation and quantitative real time polymerase chain reaction (RT-PCR)

C2C12 cells were lysed in TRIzol reagent (Thermo Fisher Scientific, Waltham, MA, USA). Total RNA was isolated following the manufacturer’s protocol. cDNA was synthesized from 1 μg of RNA using a TOPscript^TM^ RT DryMIX kit (dT18 plus) (Enzynomics, Daejeon, Korea). Real-time PCR analysis was carried out using a CFX384 C1000 Thermal Cycler (Bio-Rad, Hercules, CA, USA) using TOPreal^TM^qPCR 2 × PreMIX (SYBR Green with high ROX) (Enzynomics, Daejeon, Korea). Mouse glyceraldehyde 3-phosphate dehydrogenase (GAPDH) was used to normalize gene expression. A list of all the primers used is given in Table 1.

**Table 1:** Primers used in this study.

Gene	Sequence
*MHC Forward*	*CACCTCCACAGCACAGACAG*
*MHC Reverse*	*ACCTTGGCCATGTGATTGTT*
*Myogenin Forward*	*TACGTCCATCGTGGACAGCAT*
*Myogenin Reverse*	*TCAGCTAAATTCCCTCGCTGG*
*p62 Forward*	*GAAGCTGCCCTATACCCACA*
*p62 Reverse*	*GAGAAACCCATGGACAGCAT*
*ATG 7 Forward*	*GACCGGTCTTACCCTGCTC*
*ATG 7 Reverse*	*TGTGGTTGCTTGCTTCAGAC*
*ATG 12 Forward*	*CCATCCAAGGACTCATTGAC*
*ATG 12 Reverse*	*TTGCAGTAATGCAGGACCAG*
*ATG 14 Forward*	*GAGGGCCTTTACGTGGCTG*
*ATG 14 Reverse*	*AATAGACGAAATCACCGCTCTG*
*beclin1 Forward*	*ATGGAGGGGTCTAAGGCGTC*
*beclin1 Reverse*	*TCCTCTCCTGAGTTAGCCTCT*
*UVrag Forward*	*ACATCGCTGCTCGGAACATT*
*UVrag Reverse*	*CTCCACGTCGGATTCAAGGAA*
*PLD1 Forward*	*AGT GCT TCA GAC TTG TCC TGG GTT*
*PLD1 Reverse*	*TAT GGT AGC GTT TCG AGC TGC TGT*
*PLD2 Forward*	*TTG CGG AAG CAC TGT TTC AGT GTC*
*PLD2 Reverse*	*TTG TTC TCC GCT GTT TCT TGC CAC*
*IGF2 Forward*	*CGCTTCAGTTTGTCTGTTCG*
*IGF2 Reverse*	*AGGTAGGCGCGTCCCTCTCG*
*FST Forward*	*CTCTTCAAGTGGATGATTTTC*
*FST Reverse*	*ACAGTAGGCATTATTGGTCTG*
*GAPDH Forward*	*TCCCACTCTTCCACCTTCGA*
*GAPDH Reverse*	*CAGGAAATGAGCTTGACAAAGTTG*

### Determination of PLD activity

A phospholipase D assay kit (Sigma Aldrich, St. Louis, MO, USA) was used according to the manufacturer’s instructions.

### Immunofluorescence microscopy and quantitative analysis of myocytes

Myocytes were fixed with 10% paraformaldehyde and then washed twice with PBS. The cells were then permeabilized with 0.1% Triton-X 100 in PBS for ten minutes. Subsequent blocking was performed with 3% bovine serum albumin (BSA) in PBS for fifteen minutes. The cells were then treated with primary antibody for MHC (MF20, DSHBY) in blocking solution at 4 °C overnight. Staining for MHC was used to distinguish between differentiated and undifferentiated C2C12 cells. After overnight incubation, the cells were washed five times with PBS. Secondary antibodies (FITC-labeled) and 4′,6-diamidino-2-phenylindole (DAPI), were added to the cells for twenty minutes at room temperature. The coverslips were mounted using ProLong™ Diamond Antifade Mountant (Invitrogen, Carlsbad, CA), and images were captured using an Olympus CKX3-Houn Microscope (Olympus, Tokyo, Japan). To assess the degree of differentiation, the followings were calculated; the differentiation index (differentiated nuclei/total nuclei), fusion index (nuclei in myotubes with ≥2 nuclei/total nuclei), and average myotube size (number of nuclei/myotube). Each data point was generated by quantifying all cells in five randomly chosen microscopic fields.

### Statistical analysis

All data are presented as mean ± standard deviation (SD). Where necessary, statistical significance was determined by performing the Mann-Whitney U test. Means were calculated from the results from 4 to 9 independent experiments for all figures. P-values < 0.05 were considered statistically significant. The analysis was performed SPSS version 22 (IBM Corporation, NY, USA).

## Supplementary information

Supplementary information

## Supplementary information

is avilable for this paper at 10.1038/s41598-019-51410-7.
