# Supplementary material for: Simulated microgravity inhibits C2C12 myogenesis via phospholipase D2-induced Akt/FOXO1 regulation
Source: Sci Rep. 2019 Oct 17;9:14910. doi: 10.1038/s41598-019-51410-7 (PMC6797799; doi:10.1038/s41598-019-51410-7)
Supplement: Supplementary file 1 — Supplementary information [file 41598_2019_51410_MOESM1_ESM.docx]

**Simulated microgravity inhibits C2C12 myogenesis via**

**phospholipase D2-induced Akt/FOXO1 regulation**

Mi-Ock Baek^1,2,3,§^, Chi Bum Ahn^3, §^, Hye-Jeong Cho^2,3^, Ji-Young Choi^2,3^, Kuk Hui Son^4*^

and Mee-Sup Yoon^1, 2, 3*^

^1^Department of Health Sciences and Technology, GAIHST, ^2^ Lee Gil Ya Cancer and Diabetes Institute, ^3^Department of Molecular Medicine, School of Medicine, Gachon University, Incheon 21999, Republic of Korea, ^4^Department of Thoracic and Cardiovascular Surgery, Gachon University Gil Medical Center, College of Medicine, Gachon University, Incheon, 21565, Republic of Korea,

^§^Both authors contributed equally

Running title: Inhibition of myogenic differentiation via PLD2/Akt/FOXO1 under simulated microgravity

*Corresponding author:

Professor Kuk Hui Son, MD, PhD

Department of Thoracic and Cardiovascular Surgery

Gachon University Gil Medical Center,

College of Medicine,

Incheon, 21565, Republic of Korea

TEL: 82-32-460-3666

FAX: 82-32-899-6039

E-mail: [dr632@gilhospital.com](mailto:dr632@gilhospital.com)

Professor Mee-Sup Yoon, PhD

Department of Molecular Medicine,

School of Medicine,

Gachon University

Incheon 21999, Republic of Korea

E-mail: [msyoon@gachon.ac.kr](mailto:msyoon@gachon.ac.kr)

TEL: 82-32-899-6067

FAX: 82-32-899-6039


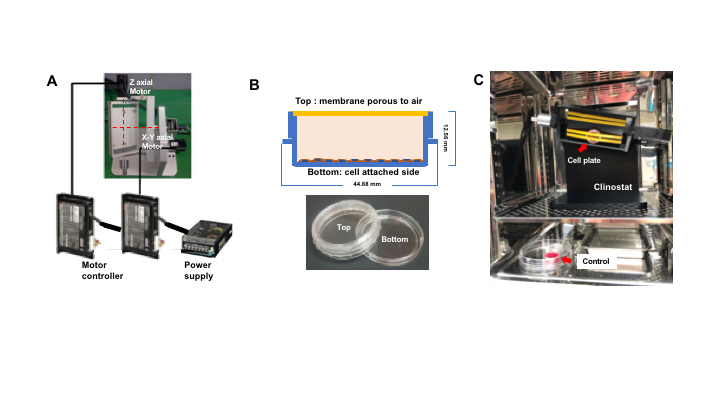


**Supplementary Figure S1 (related to Methods) 3D clinostat system**

(A) A clinostat system (3D clinostat, Shamhantech Inc., Bucheon, Korea) and (B) a PS/polyolefin based cell culture dish (SPLPermea™, SPL Life Sciences Co.) were used. (C) The dish was fixed carefully to the rotating panel of the clinostat system. The control cells (normal gravity) were plated on the same type of dish and incubated in the same incubator as the cells exposed to the SM, but did not undergo clinorotation.


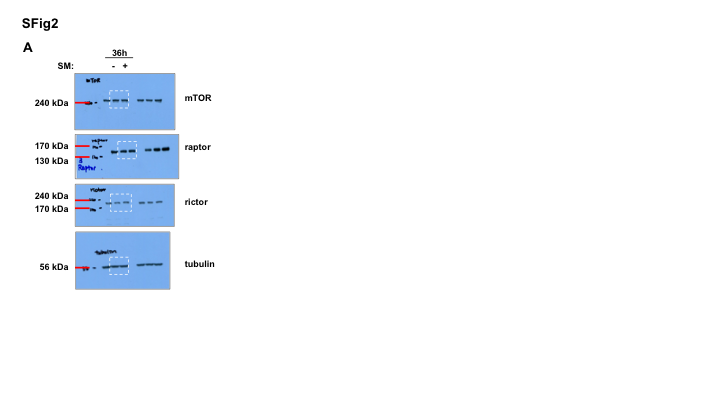


**
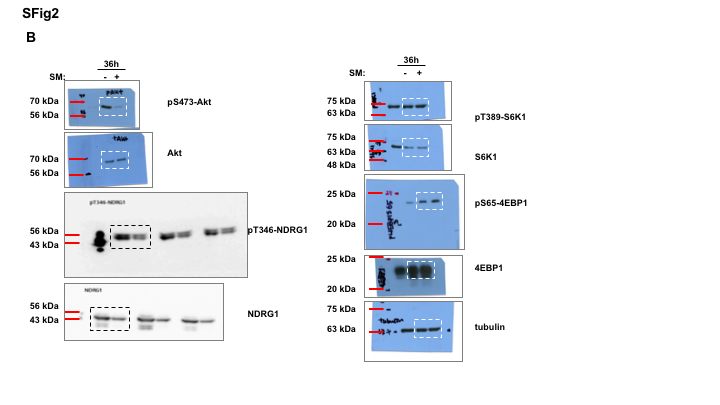
**

**Supplementary Figure S2 Original blots regarding data shown in Fig 1**

(A, B) The original blots, displayed in Fig 1A (A) and B (B) in the main manuscript, are shown here. One batch of blots was chosen to display results representatively. The dotted boxes marked the images in Fig 1. The membrane was horizontally cut into pieces, prior to first antibody incubation.


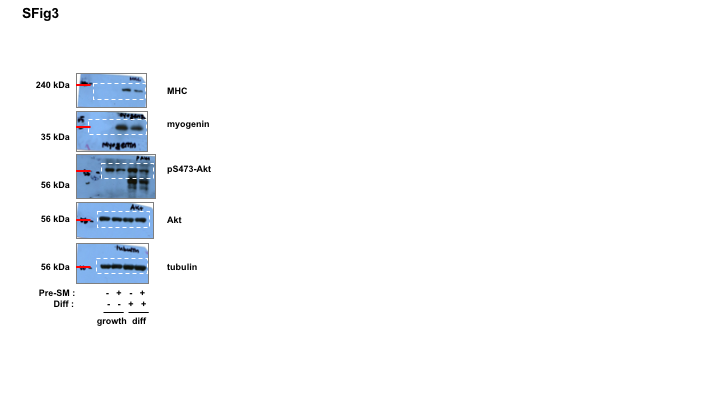


**Supplementary Figure S3 Original blots regarding data shown in Fig 2**

The original blots, displayed in Fig 2A in the main manuscript, are shown here. One batch of blots was chosen to display results representatively. The white dotted boxes marked the images in Fig 2. The membrane was horizontally cut into pieces, prior to first antibody incubation.


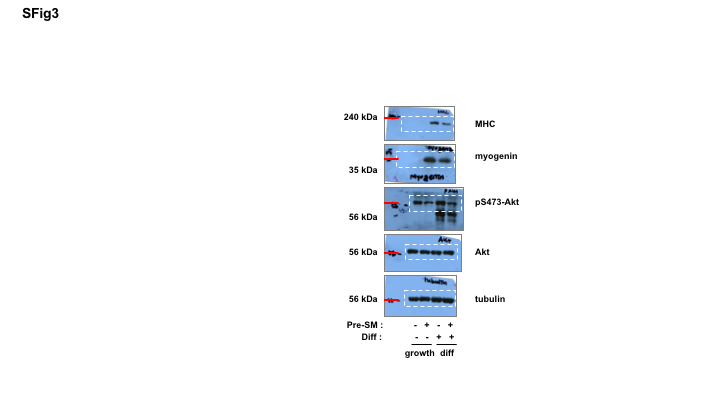


**Supplementary Figure S4 Original blots regarding data shown in Fig 3**

The original blots, displayed in Fig 3A in the main manuscript, are shown here. One batch of blots was chosen to display results representatively. The white dotted boxes marked the images in Fig 3. The membrane was horizontally cut into pieces, prior to first antibody incubation.


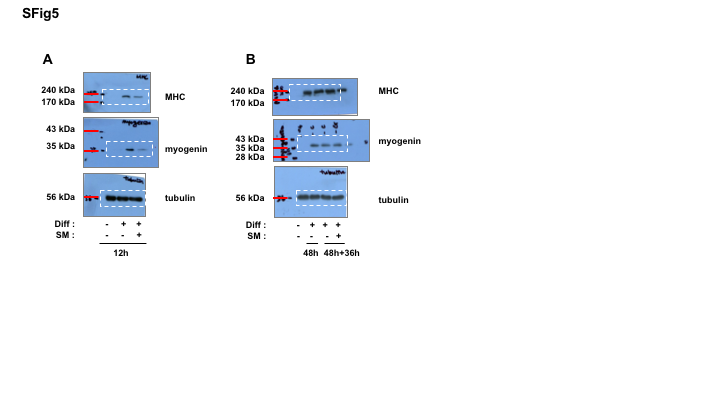


**Supplementary Figure S5 Original blots regarding data shown in Fig 4**

(A, B) The original blots, displayed in Fig 4A (A) and 4G (B) in the main manuscript, are shown here. One batch of blots was chosen to display results representatively. The white dotted boxes marked the images in Fig 4. The membrane was horizontally cut into pieces, prior to first antibody incubation.


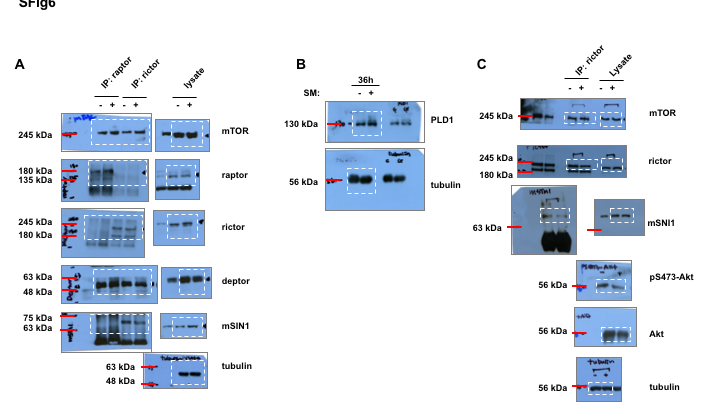


**­**

**Supplementary Figure S6 Original blots regarding data shown in Fig 5**

(A-C) The original blots, displayed in Fig 5A (A), C (B), and E (C) in the main manuscript, are shown here. One batch of blots was chosen to display results representatively. The white dotted boxes marked the images in Fig 5. The membrane was horizontally cut into pieces, prior to first antibody incubation.


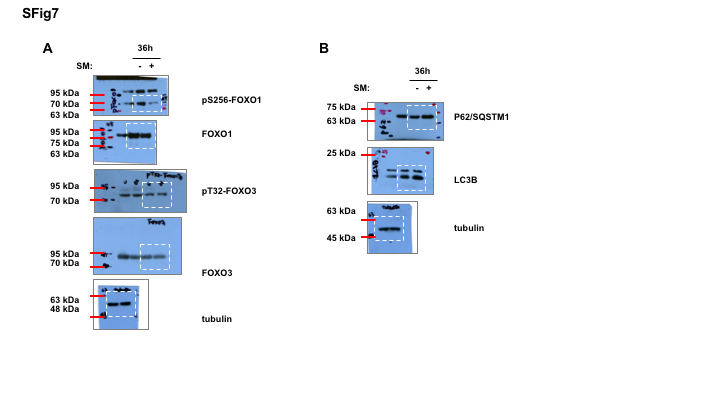


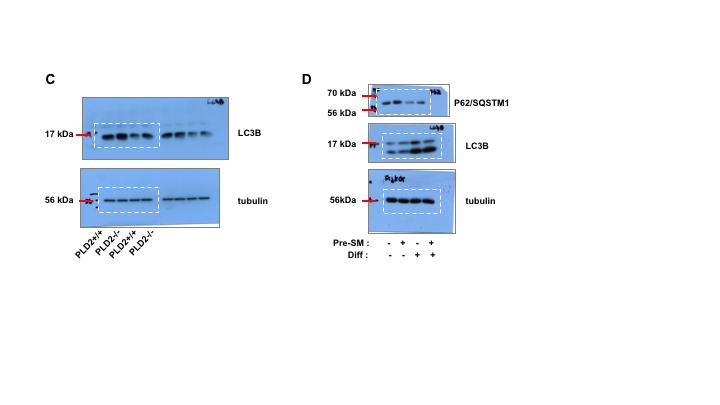


**Supplementary Figure S7 Original blots regarding data shown in Fig 6**

The original blots, displayed in Fig 6A (A), Fig. 6E (B), Fig. 6F (C), and Fig. 6G (D) in the main manuscript, are shown here. One batch of blots was chosen to display results representatively. The white dotted boxes marked the images in Fig 6. The membrane was horizontally cut into pieces, prior to first antibody incubation.
